# Phytochemical content, antioxidant and anti-inflammatory properties and wound healing effect of *Chaiturus marrubiastrum*: an in vitro and in vivo study

**DOI:** 10.1007/s10787-026-02132-6

**Published:** 2026-02-14

**Authors:** Semih Bulut, Nasif Fatih Karakuyu, Emine Sarman, Ayse Sidal, Ahmet Kahraman, Mustafa Abdullah Yilmaz, Oguz Cakir

**Affiliations:** 1 Department of Pharmacognosy, Faculty of Pharmacy, Suleyman Demirel University, Isparta, Turkey; 2 Department of Pharmacology, Faculty of Pharmacy, Suleyman Demirel University, Isparta, Turkey; 3 Department of Histology and Embryology, Faculty of Medicine, Afyonkarahisar Health Sciences University, Afyonkarahisar, Turkey; 4 Faculty of Pharmacy, Suleyman Demirel University, Isparta, Turkey; 5 Department of Molecular Biology and Genetics, Faculty of Engineering and Natural Sciences, Usak University, Usak, Turkey; 6 Department of Analytical Chemistry, Faculty of Pharmacy, Dicle University, Diyarbakir, Turkey; 7 Dicle University Science and Technology Research and Application Center, Diyarbakir, Turkey

**Keywords:** Antioxidant, Anti-inflammatory, *Chaiturus marrubiastrum*, LC-MS/MS, Pressure wounds, Rat, Wound healing

## Abstract

Medicinal plants have been traditionally used to treat wounds. The aim of this study was to investigate the antioxidant activity, wound healing effect and chemical content of the aerial parts of *Chaiturus marrubiastrum* (L.) Ehrh. ex Rchb. Pressure wound model in rats was used to evaluate the wound healing effect of the extracts. The antioxidant activity of the extract was evaluated using total antioxidant capacity, reducing power, metal chelation, DPPH and ABTS scavenging tests. The chemical profile of the extract was evaluated by LC-MS/MS analysis. The extract had 83.50 ± 0.11% and 70.73 ± 1.30% inhibition in DPPH and ABTS scavenging tests, respectively. The metal chelation capacity of the extract increased with increasing concentration. The reducing power of the extract (at 2 mg/mL) was similar to that of quercetin (3.521 ± 0.07, 3.831 ± 0.03, respectively). The most abundant substance in the extract was rosmarinic acid (165.611 mg/g extract). In vivo experiments, wound healing occurred faster in the group treated with *C. marrubiastrum* extracts in pressure wounds created on rats compared to the control groups. Histopathological analyses showed that connective tissue development and vascularization increased and inflammation decreased in this group. In addition, immunohistochemical analyses have shown that *C. marrubiastrum* extract reduces TNF-α, VEGF and caspase-3 levels, thus inhibiting inflammation and apoptosis. These findings suggest that the wound healing-accelerating effects of *C. marrubiastrum* are based on strong antioxidant and anti-inflammatory properties. It is concluded that future studies should optimize this extract for clinical use and evaluate its efficacy in humans.

**Supplementary Information:**

The online version contains supplementary material available at 10.1007/s10787-026-02132-6.

## Introduction

Damage caused by forces of different characteristics and degrees such as physical, chemical, thermal, radiation exposure, surgical causes or the condition of tissue integrity deterioration that can develop spontaneously is called a wound. Chronic wounds are wounds that are difficult to care for and heal late or do not heal (Zhao et al. 2016). Pressure wounds, which are included in the chronic wound group, are wounds that occur in certain areas of advanced age, in the epidermis and dermis, where the normal course of wound healing is slower, resistance to injury changes and the dermoepidermal connection flattens, making the tissue prone to shear and friction forces, and which can extend down to the bone, most commonly in the ischium, sacrum, trochanter major and heel displacement, in which bedridden elderly patients are bedridden for various reasons (Alderden et al. 2020). In addition, it has been determined that the incidence of pressure wounds, where immobility, albumin level and hospital stay are the most important factors, is 15–65% in patients in intensive care units in our country (Bonifant and Holloway 2019). Wound treatment is a part of health care and chronic wounds pose a serious burden to the system.

The healing process of pressure wounds consists of three basic biological phases: inflammation, proliferation, and remodeling. In the inflammation phase, vascular permeability increases in response to tissue damage, inflammatory cells migrate to the area, and necrotic tissue is cleared. During the proliferation phase, epithelialization commences with the activation of fibroblasts, angiogenesis, and the formation of granulation tissue. Finally, in the remodeling phase, collagen fibers organize, tissue strength increases, and wound contraction occurs. The effectiveness of this process is directly dependent on systemic and local factors such as pressure relief, adequate tissue perfusion, infection control, and proper nutrition (Rodrigues et al. 2019; Alsarayreh et al. 2022a, b).

Medicinal plants of the Lamiaceae family have high phenolic content. Therefore, these plants show strong anti-inflammatory and antioxidant activity. The strong antioxidant activity supports healing in wound tissues (Carović-Stanko et al. 2016; Comino-Sanz et al. 2021). Medicinal plants contribute to wound healing by reducing oxidative stress and scavenging reactive oxygen species (Joorabloo and Liu 2024; Sinha et al. 2025).

*Chaiturus marrubiastrum* (L.) Ehrh. ex Rchb. is a biennial species belonging to the Lamiaceae family, widespread in Europe and Mediterranean regions, growing in primarily in the temperature biome and waste areas. It is often called false motherwort. *Leonurus marrubiastrum* L., which grows naturally in Türkiye, has been transferred to the genus *Chaiturus* and named *C. marrubiastrum* (Gokcay 2021). In light of this information, the examination of medicinal plants in wound healing has gained importance.

This study was carried out to investigate the chemical content, antioxidant and wound healing effects of the extract prepared from the aerial parts of *C. marrubiastrum* plant which grows naturally in Türkiye. To our knowledge, this is the first time these effects will be investigated on *C. marrubiastrum*.

## Methods

### Plant material and extraction

*C. marrubiastrum* samples were collected from roadsides near the Hopa district of Artvin province in August 2023. The species was found in an altitude of about 10 m above sea level. The plant samples were identified by Prof. Dr. Ahmet Kahraman (Department of Molecular Biology and Genetics, Faculty of Engineering and Natural Sciences, Usak University, Türkiye). Voucher specimens are deposited in the GUL herbarium (code: GUL 94/46/1–1) at Suleyman Demirel University.

After the plant samples were dried, the aerial parts were ground. Methanol (≥ 99.9%, Sigma-Aldrich) was added to the aerial parts of the plant and the extract was kept on a shaker for 18 h and then the solvent was removed using a rotary evaporator (40 °C). These procedures were repeated 3 times and the extracts obtained were stored in the refrigerator (+ 4 °C) for use in experiments.

### Chemical content

#### Total phenol content

In this test, the method of Zongo et al. was used (Zongo et al. 2010). Sample was dissolved in 80% ethanol and reacted with 10% Folin-Ciocalteu. Absorbance was read at 735 nm 30 min after 7.5% Na₂CO₃ (80 µl) was added to the mixture. The result was given as mg gallic acid equivalent (GAE)/g extract.

#### Total flavonoid content

In this test, the method of Kosalec et al. was used (Kosalec et al. 2004). CH_3_COONa, C_2_H_5_OH (95%), AlCl_3_ (10%) and H₂O (560 µl) were added to the extract (1 mg/mL). The mixture was vortexed and absorbance was measured at 415 nm after 30 min.

#### LC-MS/MS analysis

In this test, the method of Yilmaz was used (Yilmaz 2020). Shimadzu-Nexera ultra-high performance liquid chromatography (UHPLC) and tandem mass spectrometry were used in the analyses. A reversed-phase 120 EC-C18 Agilent Poroshell (2.7 μm, 150 mm × 2.1 mm) column was used for analysis. The temperature of this column was determined as 40 °C. During the analysis, Solvent C (5 mM ammonium formate, water and 0.1% formic acid) and Solvent D (5 mM ammonium formate, methanol and 0.1% formic acid) systems were used. Solvent flow program: 20–100% D (0–25 min), 100% D (25–35 min), 20% D (35–45 min). Analysis parameters; injection volume: 5 µL, flow rate: 0.5 mL/min.

During mass spectrometric detection, Shimadzu LCMS-8040 tandem mass spectrometer and electrospray ionization (ESI) source was used. MS analysis parameters: nebulizing gas (N2) flow: 3 L/min, drying gas (N2) flow: 15 L/min, heat block temperature: 400 °C, interface temperature: 350 °C, DL temperature: 250 °C.

### Antioxidant activity

#### ABTS radical scavenging effect

7 mM ABTS solution and potassium persulfate (2.45 mM) were prepared with distilled water. The solutions were mixed and the resulting mixture (7.5 mL) was kept in the dark for 16 h. Phosphate buffer (pH 7.4) and ABTS solution were added to both gallic acid used as standard substance and *C. marrubiastrum* extract. The absorbance values of the mixtures were read at 734 nm (Orhan et al. 2017). The antioxidant activity of *C. marrubiastrum* extract and reference substance was determined by the following equation.

$${\mathrm{Activity}}\% = \left[ {\left( {{\mathrm{AC}} - {\mathrm{AE}}} \right)/{\mathrm{AC}}} \right] \times {\mathrm{1}}00$$

AC: Absorbance of control group, AE: Absorbance of extract.

#### DPPH radical scavenging effect

2,2-diphenyl-1-picrylhydrazyl (1 mM, DPPH) reagent was transferred onto *C. marrubiastrum* extracts and ascorbic acid used as standard substance. The final mixture was kept in the dark (25 °C) for 30 min and the absorbance of the mixture was measured at 520 nm (Jung et al. 2011). The antioxidant activity of *C. marrubiastrum* extract and reference substance was determined by the following equation.

$${\mathrm{Activity}}\% = \left[ {\left( {{\mathrm{AC}} - {\mathrm{AE}}} \right)/{\mathrm{AC}}} \right] \times {\mathrm{1}}00$$

AC: Absorbance of control group, AE: Absorbance of extract.

#### Total antioxidant capacity

*C. marrubiastrum* extract (1 mg/mL) was mixed with molybdate solution (1 mL). The mixture was kept at 90 °C for 90 min. Then the absorbance of the extract was read at 695 nm. Result was expressed as mg ascorbic acid equivalent (AAE)/g extract (Prieto et al. 1999).

#### Ferric reducing antioxidant power

Phosphate buffer (0.1 mol/L) and K_3_Fe(CN)_6_ (1%) were transferred onto both *C. marrubiastrum* extract and quercetin (standard substance), respectively. The resulting mixture was kept at 37 °C for 1 h and at the end of the time, trichloroacetic acid (10%) was added to the mixture and absorbance was measured at 700 nm. Iron (III) chloride solution (0.1%) was then added to the mixture and absorbance was measured again at 700 nm. The antioxidant effect was determined by taking the difference between these absorbance values (Oyaizu 1986).

#### Metal chelating capacity

Firstly, 2 mM iron (II) chloride was added to the extract. Then, 5 mM ferrozine was added to the *C. marrubiastrum*-FeCl_2_ mixture and the mixture was reacted at 25 °C. After 10 min, the absorbance value of the mixture was measured at 562 nm (Dinis et al. 1994). The reference substance in this test was ethylenediamintetraacetic acid (EDTA). The metal chelating capacity of *C. marrubiastrum* extract and reference substance was determined by the following equation.

$${\mathrm{Activity}}\% = \left[ {\left( {{\mathrm{AC}} - {\mathrm{AE}}} \right)/{\mathrm{AC}}} \right] \times {\mathrm{1}}00$$

AC: Absorbance of control group, AE: Absorbance of extract.

### Creaming of the extract

The obtained extract (5%) was added to the Cold cream base (Spermaceti substitute 12.5%, white beeswax 12.0%, liquid paraffin 56.0%, borax 0.5%, distilled water 19.0%) and the formulation was formed by continuous mixing. The process resulted in a 50 g cream formulation containing 5% (w/w) extract.

### In-vivo study

The study was conducted with the permission of Suleyman Demirel University Animal Experiments Local Ethics Committee Presidency, dated 15/02/2024, decision number 01/254. A total of 18 Wistar-albino female rats (250–300 g) were obtained from Suleyman Demirel University experimental animal unit.

Anesthetized rats were positioned with their dorsal region facing upwards. Before the procedure, the application area was shaved to remove hair, and after mechanical cleaning, the area was wiped with 70% isopropanol as an antiseptic and allowed to dry. To create a pressure wound model with ischemia/reperfusion (I/R), the skin on the back was gently lifted upwards with two fingers to create a skin fold. Two round magnets of the same diameter and magnetic strength were placed opposite each other on either side of this fold. The mutual attraction of the magnets subjected the skin fold to a specific pressure, restricting local blood flow and initiating the ischemia phase. The ischemia phase was applied for 4 h each day; after this, the magnets were removed, and the same area was left pressure-free for 20 h to allow the reperfusion phase to occur. This application cycle was repeated for 3 consecutive days (a total of 3 I/R cycles). During each application, the magnet positions were kept fixed to prevent displacement and to ensure that pressure was applied to the target area at the same point (Peirce et al. 2000). At the end of the three-day I/R protocol, the lesion consistent with ischemic damage developing in the area where the magnet was applied was considered a “wound area”. These rats were divided into 3 groups, 6 animals in each group. These groups are:

1. Control group: The wound area of ​​the rats in this group received no treatment for the next 7 days.
2. Extract-treated group: After the wound was created, each rat was applied 5% *C. marrubiastrum* extract dissolved in Cold cream base twice a day for 7 days.
3. Vehicle-treated group: After the wound was created, each rat was applied Cold cream base twice a day for 7 days.

After the wound was created in all experimental groups, photographs of the wounds were taken on the 1st and 7th days and their conditions were evaluated. 12 h after the last drug application, the rats were anesthetized with 90 mg/kg Ketamine and 10 mg/kg Xylazine and sacrificed by taking a high volume of blood from the inferior vena cava (surgical exsanguination) after abdominal incision, and the wound area of ​​the rats was excised including the skin and subcutaneous fascia tissue. These tissues were placed in 10% neutral formaldehyde for histopathological and immunohistochemical examination.

### Wound contraction measurements

After the wounds were created and on the last day of the experiment, photographs of the wounds were taken with a camera (iPhone 15 Pro Max) from approximately 15 cm.

Wound areas were measured by photographs taken using the ImageJ package program. In this study, the wound contraction rate was expressed as the percentage of contracted area compared to the initial wound according to the formula below (Nguyen et al. 2017).

$$\begin{gathered} Wound\;contraction\left( \% \right) \hfill \\ \;\; = \frac{{wound\;area\;at\;day\;0 - wound\;area\;at\;day\;7}}{{wound\;area\;at\;day\;0}}~ \times 100 \hfill \\ \end{gathered}$$

### Histopathological analysis

Tissues taken from each group were fixed in 10% neutral buffered formaldehyde solution, washed overnight under running water, passed through routine histological follow-up series (50%-60%-70%-80%-96%-100%), polished in xylene and embedded in paraffin blocks. 5 μm thick sections were taken from the prepared paraffin blocks. The sections were stained with Hematoxylin-Eosin (HE) and Martius Scarlet Blue (MSB) and evaluated under a light microscope. Depending on the degree and extent of the change, it was scored semi-quantitatively as 0 = absent, 1 = mild, 2 = moderate and 3 = severe (Korkmaz and Sancak 2022).

### Hematoxylin eosin (H-E)

The sections were kept at 60 °C in the oven for 60 min and then made transparent in xylene. Then, the slides were passed through 100%, 96%, 90%, 70% alcohols in order and stained in Harris Hematoxylin for 2 min. They were dipped in a 1% acid + alcohol mixture and then washed in running water for 1 min. They were kept in eosin for 2 min and passed through 70%, 80%, 96%, 100% alcohols and then transferred to xylene. They were covered with Entellan and made ready for examination (Candan et al. 2024).

### Martius scarlet blue (MSB)

The sections were kept at 60 °C in the oven for 60 min, then they were taken to xylene and deparaffinized. They were passed through 100%, 96%, 80%, 70% alcohols and then washed in running water for 1 min. They were stained with Weigert’s Iron Hematoxylin for 10 min, passed through 1% acid + alcohol and washed in running water, respectively. They were kept in 95% alcohol for 1 min. They were kept in Martius yellow and Crystal scarlet solutions for 5 min each and washed under running water, then kept in phosphotungstic acid solution for 10 min and washed under running water, and kept in aniline blue solution for 5 min. They were passed through alcohol series for 1 min each and made transparent in xylene. They were covered with Entellan and made ready for examination (Sarman et al. 2023).

### Immunohistochemical analysis

After sections 5 μm thick were cut from paraffin blocks, they were left overnight in an oven at 45 °C and then treated with xylene and alcohol series. The streptavidin-biotin complex peroxidase method was used. Primary antibodies for TNF-α (sc-52746, Santa Cruz, 1:200), Caspase-3 (sc-56053, Santa Cruz, 1:200), and VEGF (sc-57496, Santa Cruz, 1:200) were applied and incubated for 60 min. The secondary antibody (Biotinylated Goat Anti-Poliyvalent TP-125-BN, Thermo Scientific) was applied and incubated for 30 min. It was then incubated with streptavidin HRP (Horse radish peroksidaz) (TS-125-HR, Thermo Scientific) for 30 min at room temperature. The sections were washed 3 × 5 min with PBS and counterstained with Harris haematoxylin after treatment with DAB (3,3’-diaminobenzidine) (TA-125 HD, Thermo Scientific) solution. All preparations were scored at 200X magnification by selecting 10 random areas as negative (0/-), weak (1/+), moderate (2/++), and strong (3/+++) (Ozmen et al. 2024).

### Statistical analysis

For statistical expression of the comparison of experimental data between groups, Kruskal-Wallis post-hoc Dunn’s multiple comparisons test was used via GraphPad Prism v10 program. *P* < 0.05 was accepted as significant.

## Results

### Total phenol, total flavonoid contents

The extract was obtained with a yield of 10.86%, and its total phenol content was 160.87 ± 6.80 mg gallic acid equivalent (GAE)/g extract (Table 1).

**Table 1:** Results of total phenol, flavonoid content and antioxidant capacity

Extract	Yield (%)	Total phenolic content^a^ (Mean ± SD)	Total Flavonoid content^b^ (Mean ± SD)	Total antioxidant capacity^c^ (Mean ± SD)
*C. marrubiastrum*	10.86	160.87 ± 6.80	30.14 ± 1.28	168.82 ± 5.61

^a^mg GAE/g extract, ^b^mg quercetin equivalent (QE)/g extract, ^c^ mg AAE/g extract

### LC-MS/MS analysis results

**Fig. 1:**
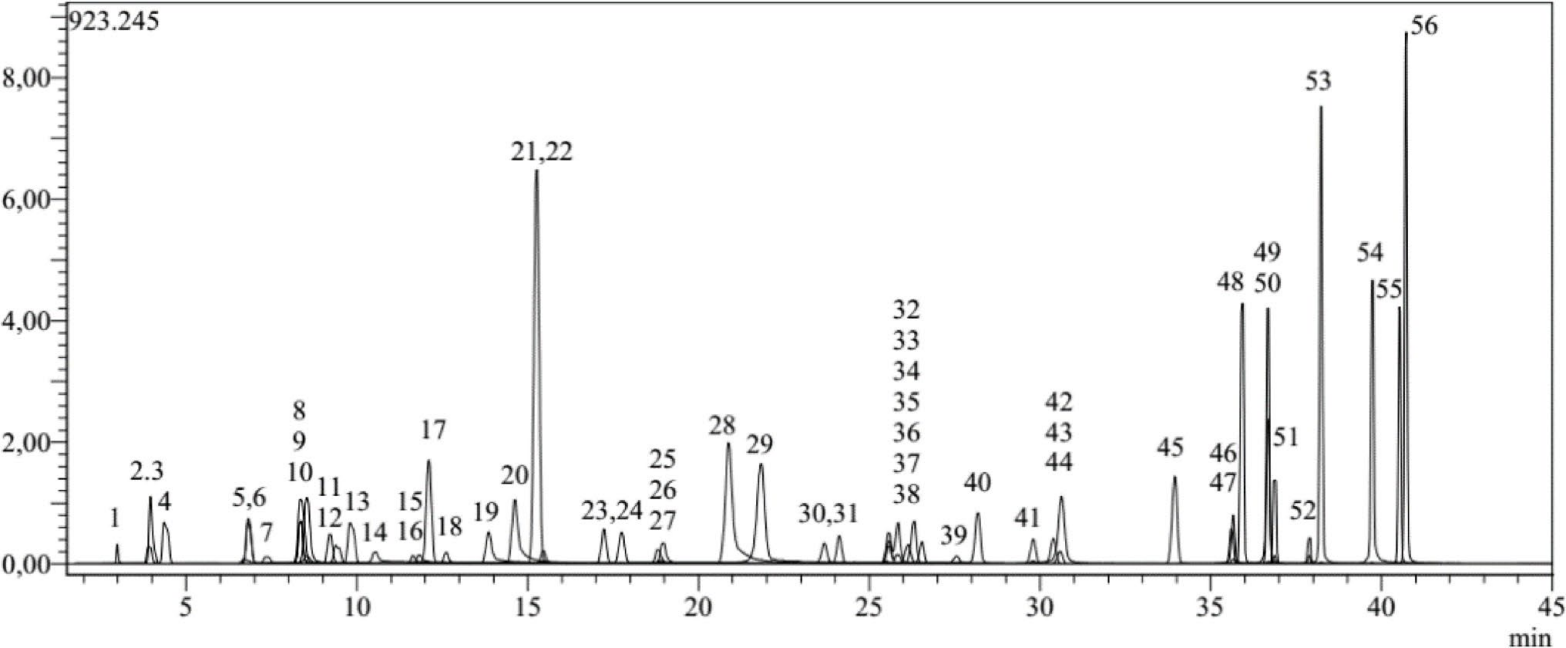
Chromatogram of standard compounds

**Fig. 2:**
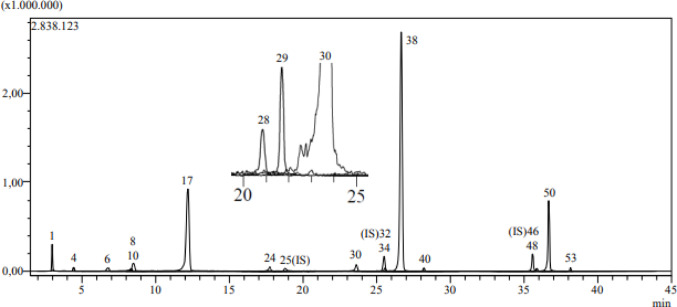
Chromatogram of the *C. marrubiastrum* extract

The phenol and flavonoid content of *C. marrubiastrum* extract was analyzed by LC-MS/MS analysis using 53 standard substances. The chromatogram of the standard substance mixture used in the analysis is shown in Fig. 1, and the chromatogram of the *C. marrubiastrum* extract is shown in Fig. 2.

According to LC-MS/MS analysis results, rosmarinic acid was the major component in the extract (165.611 mg/g extract). 16 phenolic compounds were detected in the extract, the amounts of the detected compounds are shown in Table 2. The validation parameters of 53 standard compounds are given in the supplementary table (Yilmaz 2020).

**Table 2:** Phenolic constituents of the extract

No	Compounds	*C. marrubiastrum* (mg/g extract)
1	Quinic acid	19.131
2	Fumaric aid	N.D.
3	Aconitic acid	N.D.
4	Gallic acid	0.064
5	Epigallocatechin	N.D.
6	Protocatechuic acid	1.469
7	Catechin	N.D.
8	Gentisic acid	0.415
9	Chlorogenic acid	N.D.
10	Protocatechuic aldehyde	2.156
11	Tannic acid	N.D.
12	Epigallocatechin gallate	N.D.
13	Cynarin	N.D.
14	4-OH Benzoic acid	N.D.
15	Epicatechin	N.D.
16	Vanilic acid	N.D.
17	Caffeic acid	7.807
18	Syringic acid	N.D.
19	Vanillin	N.D.
20	Syringic aldehyde	N.D.
21	Daidzin	N.D.
22	Epicatechin gallate	N.D.
23	Piceid	N.D.
24	p-Coumaric acid	0.151
25	Ferulic acid-D3-IS^h^	N.A.
26	Ferulic acid	N.D.
27	Sinapic acid	N.D.
28	Coumarin	0.052
29	Salicylic acid	0.442
30	Cyranoside	2.584
31	Miquelianin	N.D.
32	Rutin-D3-IS	N.A.
33	Rutin	N.D.
34	isoquercitrin	0.247
35	Hesperidin	N.D.
36	o-Coumaric acid	N.D.
37	Genistin	N.D.
38	Rosmarinic acid	165.611
39	Ellagic acid	N.D.
40	Cosmosiin	0.257
41	Quercitrin	N.D.
42	Astragalin	N.D.
43	Nicotiflorin	N.D.
44	Fisetin	N.D.
45	Daidzein	N.D.
46	Quercetin-D3-IS	N.A.
47	Quercetin	N.D.
48	Naringenin	0.065
49	Hesperetin	N.D.
50	Luteolin	2.753
51	Genistein	N.D.
52	Kaempferol	N.D.
53	Apigenin	0.061
54	Amentoflavone	N.D.
55	Chrysin	N.D.
56	Acacetin	N.D.

N.D.: Not detected, N.A.: Not applicable

### Antioxidant activity results

The antioxidant effect of the extract was analyzed by five methods. The extract showed strong DPPH radical scavenging activity at all concentrations and at high concentrations (1 and 2 mg/mL) had similar activity with ascorbic acid, the standard substance. Although the extract showed ABTS radical scavenging activity at all doses, this activity was weaker than that of gallic acid used as standard substance. The extract showed the strongest ABTS scavenging activity at 70.73 ± 1.3%. When the results of ferric reducing power were analyzed, the extract had very remarkable activity at a concentration of 2 mg/mL. The antioxidant activity results of the extract are shown in Table 3.

**Table 3:** Antioxidant activity test results

mg/mL	DPPH radical scavenging activity inhibition% ± SD	ABTS radical scavenging activity inhibition% ± SD	Ferric reducing power absorbance ± SD	Metal chelating capacity % ± SD
Extract				
0.25	60.05 ± 0.68***	15.87 ± 1.73**	0.502 ± 0.01***	15.42 ± 1.24***
0.5	72.03 ± 0.43***	41.33 ± 2.67***	1.101 ± 0.01***	24.18 ± 1.75***
1	80.16 ± 0.16***	45.27 ± 2.04***	2.319 ± 0.05***	36.07 ± 2.67***
2	83.50 ± 0.11***	70.73 ± 1.30***	3.521 ± 0.07***	50.42 ± 1.48***
Reference				
0.25	89.82 ± 0.28***^a^	94.29 ± 2.48***^b^	3.107 ± 0.04***^c^	99.65 ± 0.19***^d^
0.5	90.14 ± 0.19***^a^	99.49 ± 0.67***^b^	3.686 ± 0.01***^c^	99.73 ± 0.04***^d^
1	90.03 ± 0.09***^a^	97.97 ± 0.77***^b^	3.721 ± 0.10***^c^	99.78 ± 0.07***^d^
2	90.81 ± 0.34***^a^	98.22 ± 0.48***^b^	3.831 ± 0.03***^c^	99.90 ± 0.11***^d^

**p < 0.01, ***p < 0.001, ^a^ascorbic acid, ^b^gallic acid, ^c^quercetin, ^d^EDTA

### Wound contraction measurement results

**Fig. 3:**
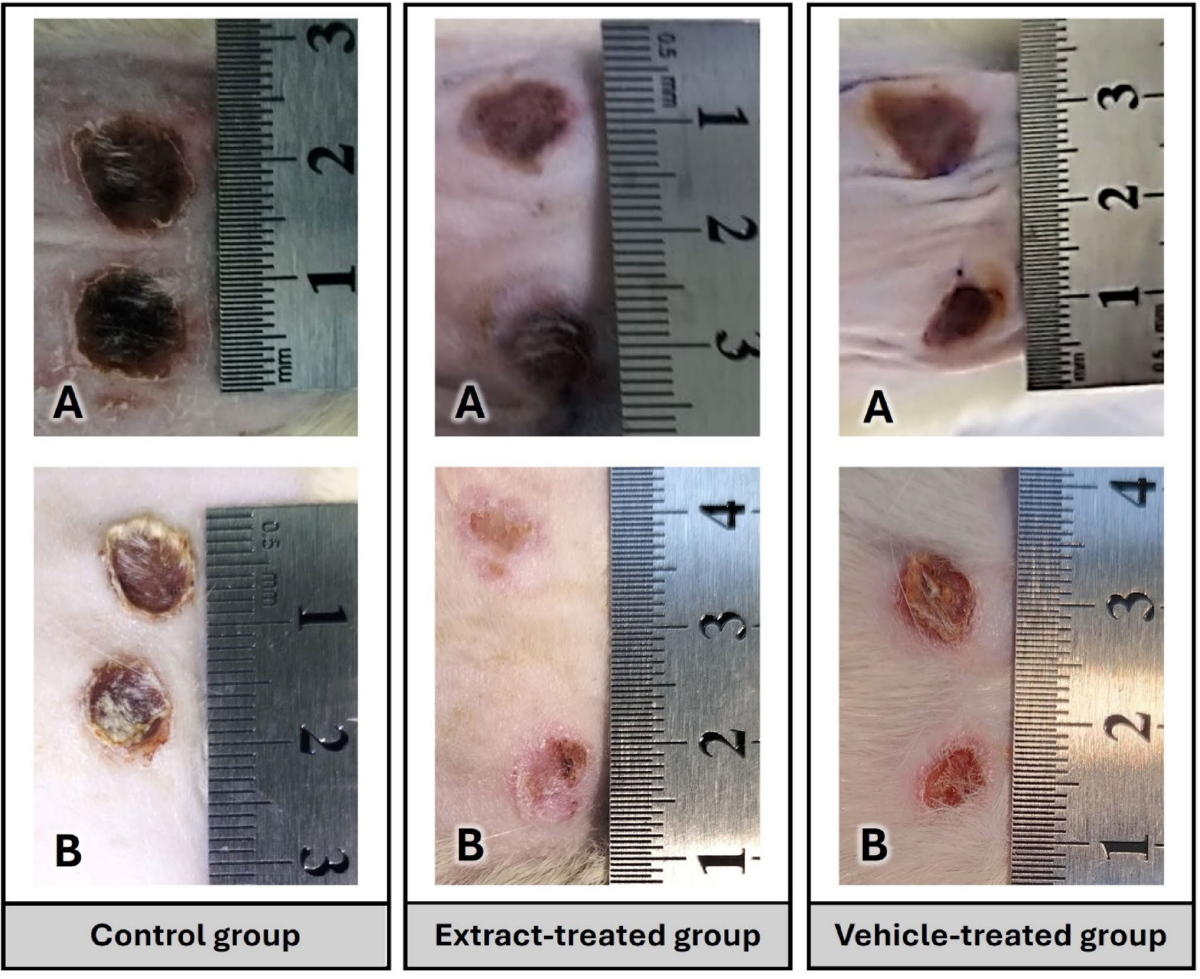
Macroscopic view of wounds at 0 and 7 days. **A** represents 0th day, **B** represents 7th day

The average wound contraction rate was calculated as 2% in the control group, 34% in the extract-treated group, and 12% in the vehicle-treated group. Sample wound photographs of the groups are also shown in Fig. 3.

### Histopathological results

**Fig. 4:**
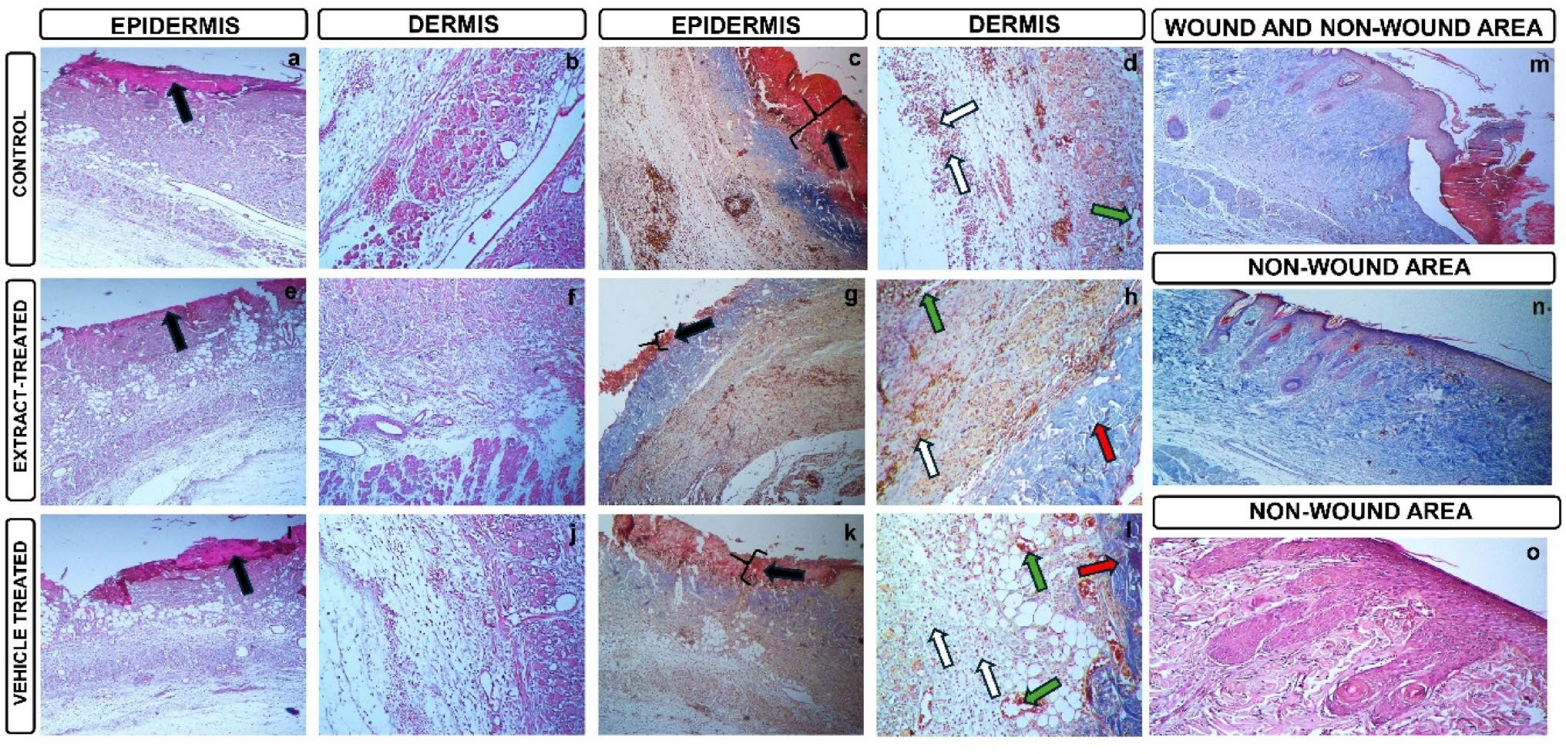
Histopathological appearances of wounds. **a**, **c**, **e**, **g**, **i**, **k**, **m**, **n** (x10; scale bar, 200 μm); **b**, **d**, **f**, **h**, **j**, **l**, **o** (x20; scale bar, 100 μm)

In the control group, the wound area is seen densely (black arrow). It is seen that the epidermis layer under the wound area has not formed at all. The damage area (black arrow) is also clearly seen with MSB, a connective tissue dye. It is seen that the collagen fibers in the dermis layer are low, inflammatory cell infiltration is seen intensely (white arrow) and angiogenesis areas are low (green arrow) (Fig. 4a, b, c, d).

In the extract-treated group, it is seen that the wound area size is reduced (black arrow) but the epidermis layer is not formed. It is determined that the collagen fibers (red arrow) and angiogenesis areas (green arrow) in the dermis layer have increased and the intensity of inflammatory cell infiltration has decreased (white arrow) (Fig. 4e, f, g, h).

In the vehicle-treated group, the wound area size is observed to be smaller (black arrow) compared to the control group. It was determined that the collagen fibers (red arrow) and angiogenesis areas (green arrow) in the dermis layer increased compared to the control group, and the intensity of inflammatory cell infiltration decreased compared to the control group (white arrow) (Fig. 4ı, j, k, l). The wound area and the non-wound area were clearly observed (Fig. 4m). In addition, the layers of the epidermis, namely st.corneum, st.lucidum, st.granulosum, st.spinosum, st.basale, hair follicles, and the dermis layer appear normal (Fig. 4n, o).

**Fig. 5:**
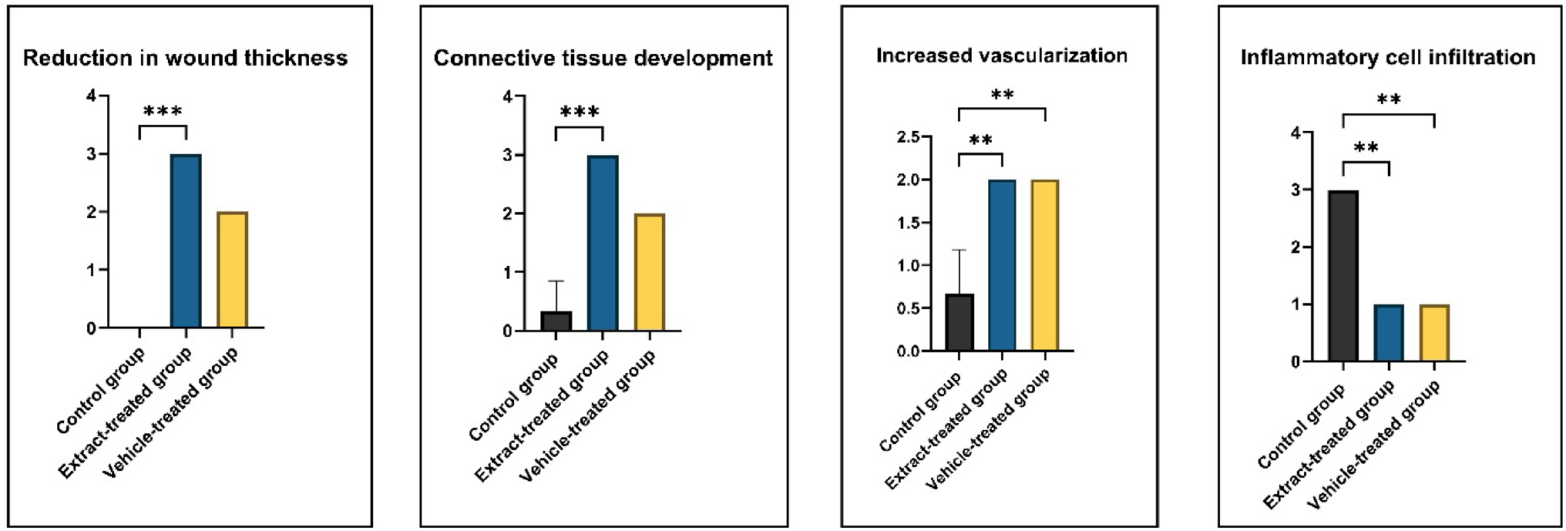
Statistical analysis results of histopathological parameters. ** represents *p* < 0.01, *** represents *p* < 0.001

The statistical analysis results of histopathological evaluations between groups are given in Fig. 5.

### Immunohistochemical results

**Fig. 6:**
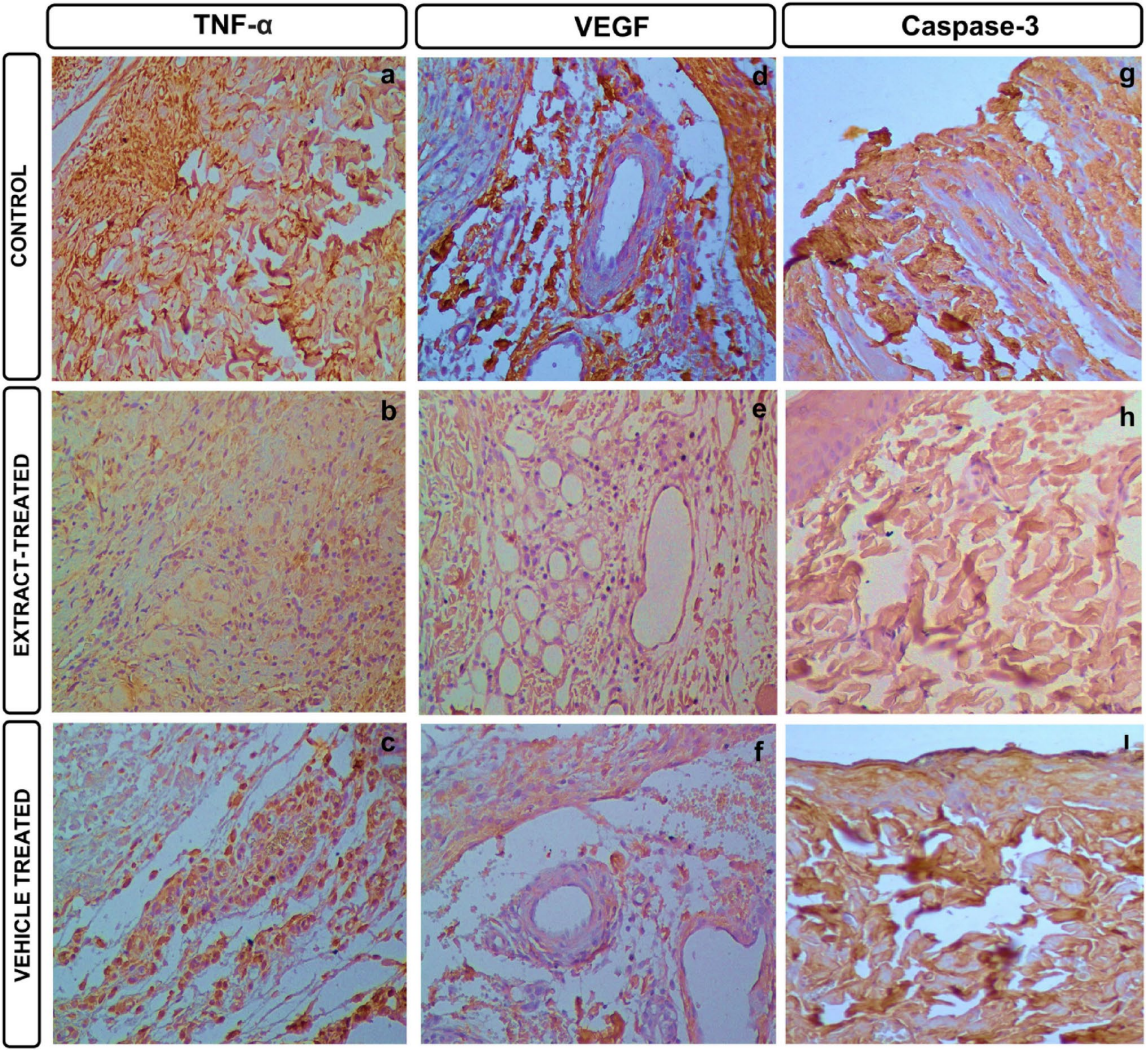
Immunohistochemical findings of wounds. **a**–**i** (x20; scale bar, 100 μm), TNF-α: Tumor necrosis factor alpha, VEGF: Vascular endothelial growth factor

**Fig. 7:**
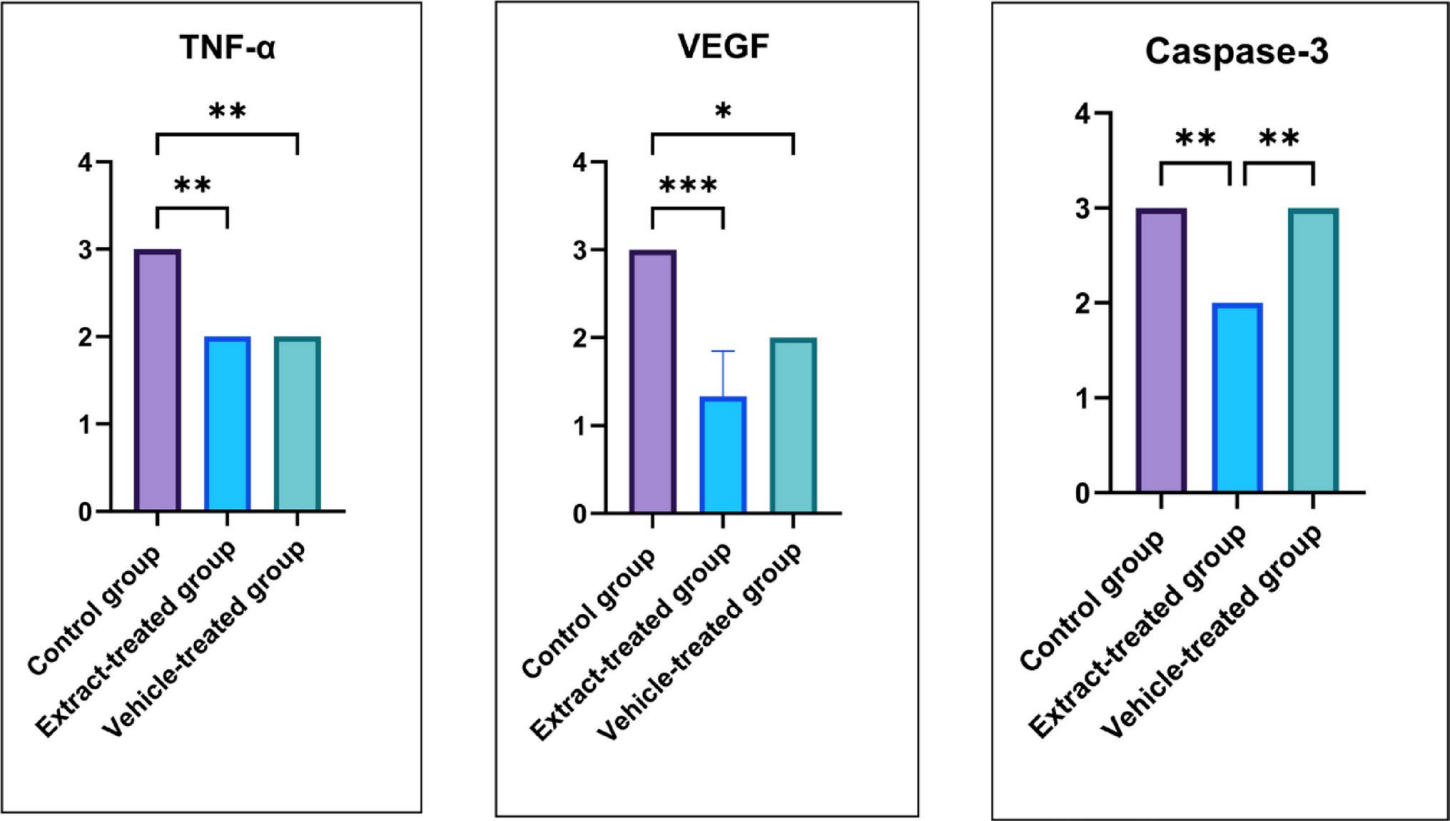
Statistical analysis results of immunohistochemical staining. ** represents *p* < 0.05, ** represents *p* < 0.01, *** represents *p* < 0.001, TNF-α: Tumor necrosis factor alpha, VEGF: Vascular endothelial growth factor

Pressure wounds created on the skin can cause inflammation, congestion, and apoptosis. TNF-α, VEGF, and Caspase-3 antibodies were studied to determine inflammatory areas, apoptotic cell density, and vasculogenesis (Fig. 6). The statistical analysis results of the immunohistochemical evaluations performed between groups are given in Fig. 7.

In the control group, TNF-α immunoreactivity of + 3 intensity is seen especially in inflammatory areas (Fig. 6a). In the extract-treated group and in the vehicle-treated group, inflammatory areas are reduced, and TNF-α immunoreactivity of + 2 intensity is remarkable (Fig. 6b and c). In the control group, VEGF immunoreactivity of + 3 intensity was observed especially around the vessels (Fig. 6d). Although immunopositive areas are seen in the extract-treated group, they are reduced and have a + 1 intensity (Fig. 6e). In the vehicle-treated group, it has a + 2 intensity (Fig. 6f). Caspase-3 immunoreactivity, an apoptotic marker, was at + 3 intensity in control and vehicle-treated groups, while it was at + 2 intensity in the extract-treated group. Immunohistochemical results showed that *C. marrubiastrum* extract reduced inflammation, apoptosis, and congestion in the wound area created in the skin.

## Discussion and conclusion

Studies have determined the presence of flavonoids and aromatic acids in *C. marrubiastrum*. Flavonoid aglycones and caffeic acid and its derivatives were found in the ethyl acetate fraction prepared from *C. marrubiastrum.* In addition, 3-caffeylquinic, 4-caffeylquinic, 5-eaffeylquinic, 1-caffeylquinie, kaemferol, quercetin and caffeic acid were isolated from *C. marrubiastrum* (Bondarenko and Litvinenko 1969). In a different study, marrubiaside and marrubialactone diterpenoids were isolated. It has also been reported that the plant contains phytol (0.04%) and sitosterol (0.05%) (Popa and Salei 1976). Marrubiastrol, desmethylmarrubiaketone, aldehydo-marrubialactone was isolated from the plant *L. marrubiastrum*, known as the synonym of *C. marrubiastrum* (Piozzi et al. 2007). Consistent with previous studies, we detected caffeic acid, but not quercetin or kaempferol. We detected high amounts of rosmarinic acid in the extract.

We found that *C. marrubiastrum* extract possesses significant antioxidant activity. This may be related to rosmarinic acid and other phenolic compounds in the extract. Rosmarinic acid’s strong antioxidant activity is closely related to its free radical scavenging and metal chelation properties (Popov et al. 2013; Khojasteh et al. 2020; Kowalczyk et al. 2024). We revealed that *C. marrubiastrum* extract (2 mg/mL) showed similar results to reference compounds in antioxidant activity tests.

Antioxidant compounds can have positive effects on wounds. Therefore, it is important to investigate antioxidant-effective sources in wound care (Comino-Sanz et al. 2021).

Anti-inflammatory sources are as important as antioxidant sources in wound healing. Rosmarinic acid promoted wound healing in the nasal mucosa in rats through its anti-inflammatory activity (Erdal et al. 2024). We also detected significant amounts of caffeic acid (7.807 mg/g extract) in *C. marrubiastrum*. Caffeic acid promoted wound healing in rats through its anti-inflammatory and antioxidant effects (Nasrullah 2023). Similar to our study, *Helianthemum canum* extract containing quinic acid and myricetin supported wound healing (Küpeli Akkol et al. 2023). Similarly, methanolic extract of *Rhus coriaria*, whose main component is myricetin 3-glucoside, reduced paw edema in diabetic rats due to its anti-inflammatory properties (Alsarayreh et al. 2024). These results indicate that plant extracts may have anti-inflammatory effects. Our results revealed that the extract had a high wound contraction rate. This may be related to its phytochemical content. *C. marrubiastrum* extract, which has both strong anti-inflammatory and antioxidant effects and a rich chemical content, may be effective in wound treatment.

It is important that the clinically demonstrated wound contraction is also supported by histological methods. Histological examination of wound sites may reveal changes such as low collagen fiber density, inflammatory cell infiltration, and low angiogenesis areas (Alsarayreh et al. 2023; Al-Qaisi et al. 2024). The decrease in wound area thickness, connective tissue development, and vascularization parameters, which were significantly higher in the extract-treated group compared to the control group, supports that wound healing is higher in the extract-treated group. At the same time, the significant decrease in inflammatory cell infiltration in the extract-treated group compared to the control group indicates that *C. marrubiastrum* extract has anti-inflammatory properties. The significantly lower TNF-α immunoreactivity in the extract-treated group compared to the control group, as shown by immunohistochemical staining methods, also supports this anti-inflammatory effect.

Hesperidin promoted wound healing by inhibiting the apoptotic pathway and reducing caspase-3 expressions in esophageal wound in rats (Durgun et al. 2023). Similarly, in our study, the significant decrease in caspase-3 levels observed in the extract-treated group compared to control and vehicle-treated groups suggests that *C. marrubiastrum* extract contributes to wound healing by inhibiting the apoptotic process accelerated by inflammation.

During the healing process following tissue injury, growth factors such as VEGF are expected to increase to some extent in the wound area. When this increase is excessive, hyperplasia and inflammation can be triggered. It can also cause complex situations, such as hypertrophic scarring during the healing process (Bao et al. 2009; Crafts et al. 2015). According to the study’s results, the fact that VEGF, which was overexpressed in the control group, was significantly lower in extract-treated and vehicle-treated groups also shows that the healing process progresses normally in these groups.

In the future, it is necessary to investigate the interaction with growth factors, cytokines, and immune cells further to investigate the effects of *C. marrubiastrum* on wound healing. Furthermore, it would be beneficial to investigate its long-term effects, especially its potential to prevent wound infections or scar formation. A limitation of this study is the absence of a standardized acute dermal toxicity test, which should be addressed in future investigations to further establish the safety profile of the extract. Furthermore, results may vary depending on the use of different extraction solvents and methods.

This research revealed that *C. marrubiastrum* may be a potential source for wound healing. The extract’s potent antioxidant and anti-inflammatory properties and rich phytochemical content are beneficial in wound healing. However, future studies should focus on optimizing the concentrations and formulations of the extract for clinical use. Furthermore, it is essential to evaluate its efficacy in human trials.

## Supplementary Information

Below is the link to the electronic supplementary material.

Supplementary Material 1

## Data Availability

All data are available by reasonable request from the corresponding author.
