# Supplementary material for: Phytochemical content, antioxidant and anti-inflammatory properties and wound healing effect of Chaiturus marrubiastrum: an in vitro and in vivo study
Source: Inflammopharmacology. 2026 Feb 14;34(3):1945–57. doi: 10.1007/s10787-026-02132-6 (PMC12995940; doi:10.1007/s10787-026-02132-6)
Supplement: Supplementary file 1 — Supplementary Material 1 [file 10787_2026_2132_MOESM1_ESM.docx]

**Table 1.** Analytical method validation parameters that belong to the LC-MS/MS method

| No | Analytes | RT*^a^* | M.I. (m/z)*^b^* | F.I. (m/z)*^c^* | Ion. mode | Equation | *r^2d^* | *RSD*%*^e^* | | Linearity Range (mg/L) | *LOD*/*LOQ* (µg/L)*^f^* | Recovery (%) | | *U^g^* | Gr. No*^i^* |
| --- | --- | --- | --- | --- | --- | --- | --- | --- | --- | --- | --- | --- | --- | --- | --- |
|  |  |  |  |  |  |  |  | Interday | Intraday |  |  | Interday | Intraday |  |  |
| 1 | Quinic acid | 3.0 | 190.8 | 93.0 | Neg | *y*=-0.0129989+2.97989*×* | 0.996 | 0.69 | 0.51 | 0.1-5 | 25.7/33.3 | 1.0011 | 1.0083 | 0.0372 | 1 |
| 2 | Fumaric aid | 3.9 | 115.2 | 40.9 | Neg | *y*=-0.0817862+1.03467*×* | 0.995 | 1.05 | 1.02 | 1-50 | 135.7/167.9 | 0.9963 | 1.0016 | 0.0091 | 1 |
| 3 | Aconitic acid | 4.0 | 172.8 | 129.0 | Neg | *y*=-0.7014530+32.9994*×* | 0.971 | 2.07 | 0.93 | 0.1-5 | 16.4/31.4 | 0.9968 | 1.0068 | 0.0247 | 1 |
| 4 | Gallic acid | 4.4 | 168.8 | 79.0 | Neg | *y*=0.0547697+20.8152*×* | 0.999 | 1.60 | 0.81 | 0.1-5 | 13.2/17.0 | 1.0010 | 0.9947 | 0.0112 | 1 |
| 5 | Epigallocatechin | 6.7 | 304.8 | 219.0 | Neg | *y*=-0.00494986+0.0483704*×* | 0.998 | 1.22 | 0.73 | 1-50 | 237.5/265.9 | 0.9969 | 1.0040 | 0.0184 | 3 |
| 6 | Protocatechuic acid | 6.8 | 152.8 | 108.0 | Neg | *y*=0.211373+12.8622*×* | 0.957 | 1.43 | 0.76 | 0.1-5 | 21.9/38.6 | 0.9972 | 1.0055 | 0.0350 | 1 |
| 7 | Catechin | 7.4 | 288.8 | 203.1 | Neg | *y*=-0.00370053+0.431369*×* | 0.999 | 2.14 | 1.08 | 0.2-10 | 55.0/78.0 | 1.0024 | 1.0045 | 0.0221 | 3 |
| 8 | Gentisic acid | 8.3 | 152.8 | 109.0 | Neg | *y*=-0.0238983+12.1494*×* | 0.997 | 1.81 | 1.22 | 0.1-5 | 18.5/28.2 | 0.9963 | 1.0077 | 0.0167 | 1 |
| 9 | Chlorogenic acid | 8.4 | 353.0 | 85.0 | Neg | *y*=0.289983+36.3926*×* | 0.995 | 2.15 | 1.52 | 0.1-5 | 13.1/17.6 | 1.0000 | 1.0023 | 0.0213 | 1 |
| 10 | Protocatechuic aldehyde | 8.5 | 137.2 | 92.0 | Neg | *y*=0.257085+25.4657*×* | 0.996 | 2.08 | 0.57 | 0.1-5 | 15.4/22.2 | 1.0002 | 0.9988 | 0.0396 | 1 |
| 11 | Tannic acid | 9.2 | 182.8 | 78.0 | Neg | *y*=0.0126307+26.9263*×* | 0.999 | 2.40 | 1.16 | 0.05-2.5 | 15.3/22.7 | 0.9970 | 0.9950 | 0.0190 | 1 |
| 12 | Epigallocatechin gallate | 9.4 | 457.0 | 305.1 | Neg | *y*=-0.0380744+1.61233*×* | 0.999 | 1.30 | 0.63 | 0.2-10 | 61.0/86.0 | 0.9981 | 1.0079 | 0.0147 | 3 |
| 13 | 1,5-dicaffeoylquinic acid | 9.8 | 515.0 | 191.0 | Neg | *y*=-0.0164044+16.6535*×* | 0.999 | 2.42 | 1.48 | 0.1-5 | 5.8/9.4 | 0.9983 | 0.9997 | 0.0306 | 1 |
| 14 | 4-OH Benzoic acid | 10.5 | 137,2 | 65.0 | Neg | *y*=-0.0240747+5.06492*×* | 0.999 | 1.24 | 0.97 | 0.2-10 | 68.4/88.1 | 1.0032 | 1.0068 | 0.0237 | 1 |
| 15 | Epicatechin | 11.6 | 289.0 | 203.0 | Neg | *y*=-0.0172078+0.0833424*×* | 0.996 | 1.47 | 0.62 | 1-50 | 139.6/161.6 | 1.0013 | 1.0012 | 0.0221 | 3 |
| 16 | Vanilic acid | 11.8 | 166.8 | 108.0 | Neg | *y*=-0.0480183+0.779564*×* | 0.999 | 1.92 | 0.76 | 1-50 | 141.9/164.9 | 1.0022 | 0.9998 | 0.0145 | 1 |
| 17 | Caffeic acid | 12.1 | 179.0 | 134.0 | Neg | *y*=0.120319+95.4610*×* | 0.999 | 1.11 | 1.25 | 0.05-2.5 | 7.7/9.5 | 1.0015 | 1.0042 | 0.0152 | 1 |
| 18 | Syringic acid | 12.6 | 196.8 | 166.9 | Neg | *y*=-0.0458599+0.663948*×* | 0.998 | 1.18 | 1.09 | 1-50 | 82.3/104.5 | 1.0006 | 1.0072 | 0.0129 | 1 |
| 19 | Vanillin | 13.9 | 153.1 | 125.0 | Poz | *y*=0.00185898+20.7382*×* | 0.996 | 1.10 | 0.85 | 0.1-5 | 24.5/30.4 | 1.0009 | 0.9967 | 0.0122 | 1 |
| 20 | Syringic aldehyde | 14.6 | 181.0 | 151.1 | Neg | *y*=-0.0128684+7.90153*×* | 0.999 | 2.51 | 0.77 | 0.4-20 | 19.7/28.0 | 1.0001 | 0.9964 | 0.0215 | 1 |
| 21 | Daidzin | 15.2 | 417.1 | 199.0 | Poz | *y*=9.45747+152.338*×* | 0.996 | 2.25 | 1.32 | 0.05-2.5 | 7.0/9.5 | 0.9955 | 1.0017 | 0.0202 | 2 |
| 22 | Epicatechin gallate | 15.5 | 441.0 | 289.0 | Neg | *y*=-0.0142216+1.06768*×* | 0.997 | 1.63 | 1.28 | 0.1-5 | 19.5/28.5 | 0.9984 | 0.9946 | 0.0229 | 3 |
| 23 | Piceid | 17.2 | 391.0 | 135/106.9 | Poz | *y*=0.00772525+25.4181*×* | 0.999 | 1.94 | 1.16 | 0.05-2.5 | 13.8/17.8 | 1.0042 | 0.9979 | 0.0199 | 1 |
| 24 | *p*-Coumaric acid | 17.8 | 163.0 | 93.0 | Neg | *y*=0.0249034+18.5180*×* | 0.999 | 1.92 | 1.43 | 0.1-5 | 25.9/34.9 | 1.0049 | 1.0001 | 0.0194 | 1 |
| 25 | Ferulic acid-D3-IS*^h^* | 18.8 | 196.2 | 152.1 | Neg | N.A. | N.A. | N.A. | N.A. | N.A. | N.A. | N.A. | N.A. | 0.0170 | 1 |
| 26 | Ferulic acid | 18.8 | 192.8 | 149.0 | Neg | *y*=-0.0735254+1.34476*×* | 0.999 | 1.44 | 0.53 | 1-50 | 11.8/15.6 | 0.9951 | 0.9976 | 0.0181 | 1 |
| 27 | Sinapic acid | 18.9 | 222.8 | 193.0 | Neg | *y*=-0.0929932+0.836324*×* | 0.999 | 1.45 | 0.52 | 0.2-10 | 65.2/82.3 | 1.0031 | 1.0037 | 0.0317 | 1 |
| 28 | Coumarin | 20.9 | 146.9 | 103.1 | Poz | *y*=0.0633397+136.508*×* | 0.999 | 2.11 | 1.54 | 0.05-2.5 | 214.2/247.3 | 0.9950 | 0.9958 | 0.0383 | 1 |

*^a^*R.T.: Retention time, *^b^*MI (*m/z):* Molecular ions of the standard analytes (m/z ratio), *^c^*FI (*m/z):* Fragment ions *^d^r*^2^: Coefficient of determination, *^e^RSD*: Relative standard deviation, *^f^LOD*/*LOQ* (µg/L): Limit of detection/quantification, *^g^U* (%): percent relative uncertainty at 95% confidence level (*k* = 2), *^h^*IS: Internal standard, *^i^*Gr. No: Represents grouping of internal standards, these numbers indicate which IS stands for which phenolic compound.

**Table 1.** Analytical method validation parameters that belong to the LC-MS/MS method (Continued)

| No | Analytes | RT*^a^* | M.I. (m/z)*^b^* | F.I. (m/z)*^c^* | Ion. mode | Equation | *r^2d^* | *RSD*%*^e^* | | Linearity Range (mg/L) | *LOD*/*LOQ* (µg/L)*^f^* | Recovery (%) | | *U^g^* | Gr. No |
| --- | --- | --- | --- | --- | --- | --- | --- | --- | --- | --- | --- | --- | --- | --- | --- |
|  |  |  |  |  |  |  |  | Interday | Intraday |  |  | Interday | Intraday |  |  |
| 29 | Salicylic acid | 21.8 | 137.2 | 65.0 | Neg | *y*=0.239287+153.659*×* | 0.999 | 1.48 | 1.18 | 0.05-2.5 | 6.0/8.3 | 0.9950 | 0.9998 | 0.0158 | 1 |
| 30 | Cynaroside | 23.7 | 447.0 | 284.0 | Neg | *y*=0.280246+6.13360*×* | 0.997 | 1.56 | 1.12 | 0.05-2.5 | 12.1/16.0 | 1.0072 | 1.0002 | 0.0366 | 2 |
| 31 | Miquelianin | 24.1 | 477.0 | 150.9 | Neg | *y*=-0.00991585+5.50334*×* | 0.999 | 1.31 | 0.95 | 0.1-5 | 10.6/14.7 | 0.9934 | 0.9965 | 0.0220 | 2 |
| 32 | Rutin-D3-IS*^h^* | 25.5 | 612.2 | 304.1 | Neg | N.A. | N.A. | N.A. | N.A. | N.A. | N.A. | N.A. | N.A. | N.A. | 2 |
| 33 | Rutin | 25.6 | 608.9 | 301.0 | Neg | *y*=-0.0771907+2.89868*×* | 0.999 | 1.38 | 1.09 | 0.1-5 | 15.7/22.7 | 0.9977 | 1.0033 | 0.0247 | 2 |
| 34 | isoquercitrin | 25.6 | 463.0 | 271.0 | Neg | *y*=-0.111120+4.10546*×* | 0.998 | 2.13 | 0.78 | 0.1-5 | 8.7/13.5 | 1.0057 | 0.9963 | 0.0220 | 2 |
| 35 | Hesperidin | 25.8 | 611.2 | 449.0 | Poz | *y*=0.139055+13.2785*×* | 0.999 | 1.84 | 1.35 | 0.1-5 | 19.0/26.0 | 0.9967 | 1.0043 | 0.0335 | 2 |
| 36 | *o*-Coumaric acid | 26.1 | 162.8 | 93.0 | Neg | *y*=0.00837193+11.2147*×* | 0.999 | 2.11 | 1.46 | 0.1-5 | 31.8/40.4 | 1.0044 | 0.9986 | 0.0147 | 1 |
| 37 | Genistin | 26.3 | 431.0 | 239.0 | Neg | *y*=1.65808+7.57459*×* | 0.991 | 2.01 | 1.28 | 0.1-5 | 14.9/21.7 | 1.0062 | 1.0047 | 0.0083 | 2 |
| 38 | Rosmarinic acid | 26.6 | 359.0 | 197.0 | Neg | *y*=-0.0117238+8.04377*×* | 0.999 | 1.24 | 0.86 | 0.1-5 | 16.2/21.2 | 1.0056 | 1.0002 | 0.0130 | 1 |
| 39 | Ellagic acid | 27.6 | 301.0 | 284.0 | Neg | *y*=0.00877034+0.663741*×* | 0.999 | 1.57 | 1.23 | 0.4-20 | 56.9/71.0 | 1.0005 | 1.0048 | 0.0364 | 1 |
| 40 | Cosmosiin | 28.2 | 431.0 | 269.0 | Neg | *y*=-0.708662+8.62498*×* | 0.998 | 1.65 | 1.30 | 0.1-5 | 6.3/9.2 | 0.9940 | 0.9973 | 0.0083 | 2 |
| 41 | Quercitrin | 29.8 | 447.0 | 301.0 | Neg | *y*=-0.00153274+3.20368*×* | 0.999 | 2.24 | 1.16 | 0.1-5 | 4.8/6.4 | 0.9960 | 0.9978 | 0.0268 | 2 |
| 42 | Astragalin | 30.4 | 447.0 | 255.0 | Neg | *y*=0.00825333+3.51189*×* | 0.999 | 2.08 | 1.72 | 0.1-5 | 6.6/8.2 | 0.9968 | 0.9957 | 0.0114 | 2 |
| 43 | Nicotiflorin | 30.6 | 592.9 | 255.0/284.0 | Neg | *y*=0.00499333+2.62351*×* | 0.999 | 1.48 | 1.23 | 0.05-2.5 | 11.9/16.7 | 0.9954 | 1.0044 | 0.0108 | 2 |
| 44 | Fisetin | 30.6 | 285.0 | 163.0 | Neg | *y*=0.0365705+8.09472*×* | 0.999 | 1.75 | 1.19 | 0.1-5 | 10.1/12.7 | 0.9980 | 1.0042 | 0.0231 | 3 |
| 45 | Daidzein | 34.0 | 253.0 | 223.0 | Neg | *y*=-0.0329252+6.23004*×* | 0.999 | 2.18 | 1.73 | 0.1-5 | 9.8/11.6 | 0.9926 | 0.9963 | 0.0370 | 3 |
| 46 | Quercetin-D3-IS*^h^* | 35.6 | 304.0 | 275.9 | Neg | N.A. | N.A. | N.A. | N.A. | N.A. | N.A. | N.A. | N.A. | N.A. | 3 |
| 47 | Quercetin | 35.7 | 301.0 | 272.9 | Neg | *y*=+0.00597342+3.39417*×* | 0.999 | 1.89 | 1.38 | 0.1-5 | 15.5/19.0 | 0.9967 | 0.9971 | 0.0175 | 3 |
| 48 | Naringenin | 35.9 | 270.9 | 119.0 | Neg | *y*=-0.00393403+14.6424*×* | 0.999 | 2.34 | 1.69 | 0.1-5 | 2.6/3.9 | 1.0062 | 1.0020 | 0.0392 | 3 |
| 49 | Hesperetin | 36.7 | 301.0 | 136.0/286.0 | Neg | *y*=+0.0442350+6.07160*×* | 0.999 | 2.47 | 2.13 | 0.1-5 | 7.1/9.1 | 0.9998 | 0.9963 | 0.0321 | 3 |
| 50 | Luteolin | 36.7 | 284.8 | 151.0/175.0 | Neg | *y*=-0.0541723+30.7422*×* | 0.999 | 1.67 | 1.28 | 0.05-2.5 | 2.6/4.1 | 0.9952 | 1.0029 | 0.0313 | 3 |
| 51 | Genistein | 36.9 | 269.0 | 135.0 | Neg | *y*=-0.00507501+12.1933*×* | 0.999 | 1.48 | 1.19 | 0.05-2.5 | 3.7/5.3 | 1.0069 | 1.0012 | 0.0337 | 3 |
| 52 | Kaempferol | 37.9 | 285.0 | 239.0 | Neg | *y*=-0.00459557+3.13754*×* | 0.999 | 1.49 | 1.26 | 0.05-2.5 | 10.2/15.4 | 0.9992 | 0.9990 | 0.0212 | 3 |
| 53 | Apigenin | 38.2 | 268.8 | 151.0/149.0 | Neg | *y*=0.119018+34.8730*×* | 0.998 | 1.17 | 0.96 | 0.05-2.5 | 1.3/2.0 | 0.9985 | 1.0003 | 0.0178 | 3 |
| 54 | Amentoflavone | 39.7 | 537.0 | 417.0 | Neg | *y*=0.727280+33.3658*×* | 0.992 | 1.35 | 1.12 | 0.05-2.5 | 2.8/5.1 | 0.9991 | 1.0044 | 0.0340 | 3 |
| 55 | Chrysin | 40.5 | 252.8 | 145.0/119.0 | Neg | *y*=-0.0777300+18.8873*×* | 0.999 | 1.46 | 1.21 | 0.05-2.5 | 1.5/2.8 | 0.9922 | 1.0050 | 0.0323 | 3 |
| 56 | Acacetin | 40.7 | 283.0 | 239.0 | Neg | *y*=-0.559818+163.062*×* | 0.997 | 1.67 | 1.28 | 0.02-1 | 1.5/2.5 | 0.9949 | 1.0011 | 0.0363 | 3 |

*^a^*R.T.: Retention time, *^b^*MI (*m/z):* Molecular ions of the standard analytes (m/z ratio), *^c^*FI (*m/z):* Fragment ions *^d^r^2^*: Coefficient of determination, *^e^RSD*: Relative standard deviation, *^f^LOD*/*LOQ* (µg/L): Limit of detection/quantification, *^g^U* (%): percent relative uncertainty at 95% confidence level (*k* = 2), *^h^*IS: Internal standard, *^i^*Gr. No: Represents grouping of internal standards, these numbers indicate which IS stands for which phenolic compound.
